# A Comparative Analysis of Folate and Mineral Contents in Freshly Squeezed and Commercial 100% Orange Juices Available in Europe

**DOI:** 10.3390/nu16213605

**Published:** 2024-10-24

**Authors:** Montaña Cámara, Laura Domínguez, Sonia Medina, Pedro Mena, Cristina García-Viguera

**Affiliations:** 1 Nutrition and Food Science Department, Pharmacy Faculty, Complutense University of Madrid (UCM), Plaza Ramón y Cajal, s/n, 28040 Madrid, Spain; ladoming@ucm.es; 2 Lab. Fitoquímica y Alimentos Saludables (LabFAS), CSIC, CEBAS, Campus Universitario Espinardo 25, 30100 Murcia, Spain; smescudero@cebas.csic.es (S.M.); cgviguera@cebas.csic.es (C.G.-V.); 3 Human Nutrition Unit, Department of Food and Drug, University of Parma, 43124 Parma, Italy

**Keywords:** plant foods, bioactive compounds, nutrients, shelf life, storage, nutrition claims

## Abstract

Background: Orange juices are widely known for their organoleptic characteristics and potential health benefits derived from their nutritional and functional composition. Objectives: The aim of this work was to provide comprehensive, up-to-date information on the content of folates and some minerals (Ca, K, Mg, Fe, Mn, and Zn) present in different fresh and commercial orange juices available in the European market, including juices from concentrate (FC) and not from concentrate (NFC). Methods: A total of twenty-five juice samples were selected, comprising the most purchased types of commercial juices from France, the United Kingdom (UK), Germany, and Spain, and four fresh squeezed juices (FSJ) made from Spanish oranges. In FSJ, nutrient stability during storage time (0–48 h) was also assessed. Results: Folate concentration was highly variable between commercial samples, with higher contents in FSJ and NFC samples, followed by FC juices. Regarding mineral content, FSJ showed significant differences with FC (except for Mg) and NFC juice samples (except for Ca and Mg), while FC and NFC had similar mineral profiles, except for Ca. Storage time had no significant impact on FSJ folates and minerals. Conclusions: Among commercial juices, the NFC category generally showed the highest content of folates, K, Mg, and Fe, whereas FC juices showed the highest contents of Ca, Mn, and Zn. Some commercial juices met the legal use conditions for a “Source of folate” claim, whereas both commercial and fresh juices met the conditions for a “Source of potassium” claim, according to European and UK regulations.

## 1. Introduction

Among the various types of citrus fruits, oranges (*Citrus sinensis*) are the most extensively cultivated, comprising over half of the world citrus production and being the most frequently traded [1]. In Europe, citrus production was projected to be 9.9 million tons, with 85% coming from the oranges and mandarins/tangerines categories [2]. Oranges have significant use and acceptance as a food source for humans, also including orange juices.

Oranges and their juices are widely known for their characteristic taste and aroma and potential health benefits derived from their nutritional and functional characteristics [3]. Oranges are abundant in bioactive compounds, such as folic acid, minerals, and polyphenols, all of which are crucial for overall nutritional and health status [4]. Folic acid plays a crucial role in neural tube closure by influencing processes such as nucleotide biosynthesis and methylation reactions [5,6,7]. In addition, folates are important in the synthesis of amino acids, in the process of cell division, and in blood formation, with a special incidence during pregnancy when its requirements are increased [8]. On the other hand, macro- and micro-minerals serve as cofactors in numerous enzymatic reactions and various physiological functions. Calcium (Ca) and magnesium (Mg) are vital for the nervous and muscular systems and play a key role in bone formation. Mg also plays a crucial role in glucose metabolism, and low serum levels of this mineral have been linked to different disorders and chronic diseases, such as diabetes mellitus [9]. Copper (Cu) and iron (Fe) are essential for the circulatory system, with Cu involved in hemoglobin synthesis and enhancing iron absorption from the digestive tract. Fe is one essential micronutrient for several bodily functions, including energy metabolism, red blood cell and hemoglobin formation, and oxygen transport in the body, and it is involved in the process of cell division and cell death. Iodine (I) is also considered an important element for its crucial role in the synthesis of thyroid hormones, which are vital for brain and central nervous system development, and selenium (Se) is crucial for iodine metabolism within the thyroid. Finally, zinc (Zn) is important for cell reproduction, sperm and testosterone production, as well as growth and development in children [10].

Knowledge of the composition of these bioactive compounds in foods, particularly orange juices, is essential for the correct application of legislation on food information provided to the consumer concerning its nutritional and/or health properties. In this context, European Regulation (EC) Nº 1924/2006 establishes the conditions to consider a food product as a source of vitamins (as folates) and minerals, and Regulation (EU) Nº 1169/2011 indicates its Nutrient Reference Value (NRV) for folates and certain minerals [11,12].

In this regard, a recent study by our research group examined the vitamin C and flavanone content of commercial juices compared with freshly squeezed juices made from “Navelina” oranges. Results showed that flavanones and vitamin C levels were constant among countries and processing systems. In addition, orange juices preserved their bioactive compounds during a typical period of shelf life [13]. The present work builds on the former by providing comprehensive, up-to-date information on the content of folates and some minerals (Ca, K, Mg, Fe, Mn, and Zn) present in fresh and commercial orange juices available on the European market.

## 2. Materials and Methods

### 2.1. Samples

A total of 25 orange juice samples were included in this analysis. The majority (21/25, 84%) were commercial 100% orange juice samples purchased from local markets in 4 different countries (France, United Kingdom [UK], Germany, and Spain), whereas 4 samples corresponded to fresh orange juices made according to the procedures described by Salar et al. (2024) [13].

Regarding the commercial juices, market research was conducted to identify the orange juice products with the highest market share in each of the 4 targeted countries. A total of 6 samples were selected from the UK market (since one product was available with or without orange cells), whereas 5 commercial orange juices were selected from the French, German, and Spanish markets. The present study included both chilled “not from concentrate” (NFC) orange juices and ambient “from concentrate” (FC) orange juices. All commercial samples were transported at the recommended temperature via courier (3 bottles per sample) to the LabFAS, CEBAS-CSIC (Murcia, Spain).

Furthermore, four sets of Navelina oranges were freshly harvested and sourced from the following Spanish companies (AMC Group, Murcia; Riverbend, Murcia; and ZUVAMESA (ZVM), Valencia) to make freshly squeezed juices (FSJ) under controlled laboratory conditions using an electric citrus juicer (Orbegozo EP 2210, Murcia, Spain). A total of 20 kg of fresh oranges per company were used to obtain the freshly squeezed juices (FSJ).

Shelf-life assessment tests were performed by analyzing the content of different bioactive compounds (folates and minerals) at 0-, 12-, 24-, and 48-h post-juicing and stored at 5 °C. This was performed to assess the rate of degradation of these bioactive compounds. The timeframe was chosen to reflect the typical duration of FSJ storage in a home refrigerator [13]. Fresh orange juices were extracted in triplicate. Table 1 describes the orange juices analyzed in this study.

Table 1 describes the orange juices analyzed in this study.

### 2.2. Methodology

#### 2.2.1. Determination of Folate Concentration

The determination of folates in the form of 5-methyl-tetrahydrofolic acid (5-MTHF) in the selected orange juices consisted of extraction in phosphate buffer (Na_2_HPO_4_ 2H_2_O, 0.1 M, pH = 7, containing 0.1% L-ascorbic acid), heat treatment (100 °C), and centrifugation [14]. Following that, an enzymatic treatment with rat serum and incubation (at 37 °C for 2 h) were carried out. Finally, juice samples were subjected to centrifugation, filtration (0.2 µm), and quantification by liquid chromatography coupled with tandem mass spectrometry (LC-MS/MS) according to PNT-LACC/FQ 254, Analiza Calidad (Madrid, Spain) [15,16]. Centrifugation was performed during 15 min at 4000 rpm and room temperature using Unicen 21 equipment (supplied by Ortoalresa Álvarez Redondo S.A, Madrid, Spain). The equipment and chromatographic parameters used in the determination of folate concentration are described as follows: liquid chromatograph coupled to a triple quadrupole mass spectrometer (Agilent 6460 model, Agilent Technology, Santa Clara, CA, USA), C18 column (2.1 × 100 mm, 1.8 µm), flow rate 0.2 mL/min, and gradient mobile phase (A: H_2_O acidified with 0.2% formic acid; B: MeOH acidified with 0.2% formic acid). The preparation of a calibration curve was necessary for the folate quantification, and it was daily prepared from a standard solution of 5-MTHF (supplied by Supelco, Darmstadt, Germany, ref. 47866) in the same phosphate buffer used in the extraction process. Results were expressed as µg 5-methyl-tetrahydrofolic acid (5-MTHF)/100 mL orange juice.

#### 2.2.2. Determination of Mineral Concentration

Minerals (Ca, K, Mg, Fe, Mn, and Zn) of orange juice samples were subject to an acid digestion (HNO_3_–HClO_4_, 2:1, *v*/*v*). The extract aliquot resulting from this digestion was diluted with LaCl_3_ + CsCl, and mineral content was determined using an inductively coupled plasma (ICP) spectrometer (OES Thermo ICAP 6000 SERIES^®^; ThermoElectron Corp., Franklin, MA, USA), according to Domínguez-Perles et al. (2010) [17].

## 3. Results and Discussion

### 3.1. Folate Content in Commercial and Fresh Orange Juices

Since folate degradation may occur during industrial production and storage of commercial orange juice, in the present study, the folate content of the commercial and fresh orange juice samples was assessed.

Folate concentration was highly variable among commercial samples, with several differences reaching statistical significance. Higher content corresponded to fresh juices (FSJ) and NFC samples, without statistical differences among them, while the lowest content corresponded to orange juice samples from concentrate (FC), with significant differences in comparison to the other juices, both FSJ and NFC (Table 2).

Considering the purchasing location, juices sold in France had the highest values (19.55 µg 5-MTHF/100 mL on average), mostly NFC, while those sold in Germany had the lowest (4.75 µg 5-MTHF/100 mL on average), being all of them FC. In the case of the individual commercial orange juices (FC) sold in Germany, no significant differences (*p* > 0.05) were found between samples except for GJ5C. Samples purchased in Spain were NFC and FC, with no significant differences (*p* > 0.05) among them except for SJ5C. Interestingly, the highest folate contents were found in two fresh orange juice samples, SFSJ4 (25.51 µg 5-MTHF/100 mL) and SFSJ1 (23.86 µg 5-MTHF/100 mL), followed by the commercial orange juices purchased in France (17.25–23.19 µg 5-MTHF/100 mL) and the UK (13.45–21.13 µg 5-MTHF/100 mL). These values are in the range reported by Delchier et al. (2016) and Öhrvik et al. (2008) [18,19].

**Figure 1:**
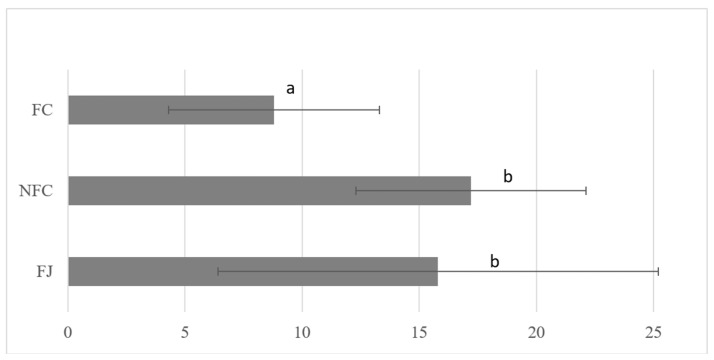
Mean folic acid (5-MTHF) content in fresh (FJ) and commercial non from concentrate (NFC) and from concentrate (FC) orange juice samples. According to Fisher’s least significant difference (LSD) procedure, values (the mean of all samples from the same type of juice) followed by the same letter were not significantly different (*p* > 0.05).

The average folate content across the three different types of orange juice (FSJ, NFC, and FC) was 12.75 µg 5-MTHF/100 mL and median 13.48 µg 5-MTHF/100 mL (Figure 1). Orange juices with the lowest folate concentrations tended to be commercial FC samples purchased from Germany (range: 3.850–6.790 µg 5-MTHF/100 mL) and one FSJ sample (SFSJ2), which contained 6.025 µg 5-MTHF/100 mL. The lower folate levels in the FC samples may reflect different types of packaging, higher storage temperatures, and longer shelf life. These factors were not standardized in the sampling methodology.

#### 3.1.1. Folate Stability in Fresh Orange Juices

Folic acid is a hydrosoluble vitamin with a high degradation rate that depends on the storage conditions (temperature, light exposure) and storage time. To verify whether folic acid concentration is stable in fresh orange juices during a reasonable shelf-life period, a study of folate stability was performed in these samples at four time points (t = 0, t = 12 h, t = 24 h, and t = 48 h). As can be seen in Table 3, storage time had not a significant effect (*p* > 0.05) on folic acid content in the following samples: SFSJ1 juices (t0 h vs. t48 h) (23.86 vs. 18.90 µg 5-MTHF/100 mL in 48 h), SFSJ2 samples (6.02 vs. 5.66 µg 5-MTHF/100 mL), and SFSJ3 juices (7.79 vs. 6.92 µg 5-MTHF/100 mL). However, the SFSJ4 sample showed significant differences (*p* < 0.01) at 12 h (25.51 vs. 23.31 µg 5-MTHF/100 mL).

#### 3.1.2. Orange Juice Contribution to Folate Requirements

Consumption of the analyzed orange juices could contribute to the achievement of folate daily requirements established for different target groups following the scientific reports published by the European Food Safety Authority (EFSA) in 2017 (last amendment in 2019) [20]. As it can be deduced from the obtained results (Table 4), commercial orange juices sold in France could make the largest contribution to folate daily requirements, followed by orange juices sold in the UK, Spain, and Germany.

Regarding population sub-groups, children are the group most likely to benefit. Daily consumption of one glass of commercial orange juice (200 mL) analyzed in the present study could provide 7.9–32.6% of the folate daily requirements for children aged 1 to 3 years old; 6.3–26.1% for those between 4 and 5 years; and 4.8–19.6% and 3.5–14.5% in the case of children aged 6–9 years and 10–13 years old, respectively. Teenagers (14–19 years old) and the general adult population (20–70 years old) would also benefit from the intake of folate from commercial orange juices. Considering those samples with the highest content of folates (France and UK), it can be concluded that almost 10% of folate requirements could be fulfilled in these groups from one daily serving of orange juice.

Regarding fresh orange juice samples (SFSJ1, SFSJ2, SFSJ3, and SFSJ4), the contribution of one glass (200 mL) at t0 was much higher than the commercial samples (Table 4). For instance, 10.0–42.5% and 8.0–34.0% of folate daily requirements in toddlers between 1 and 3 years and 4 and 5 years old could be met with the consumption of these fresh orange juices, respectively. In relation to children between 6 and 9 years and 10 and 13 years old, folate contributions of 25.5 and 18.9% could be reached by consuming SFSJ4, the fresh orange juice with the highest content of folic acid at 0. Teenagers (14–19 years old) and the general population (20–70 years old) would also benefit from the intake of these fresh orange juices, with a folate daily contribution of 15.5%, whereas pregnant and lactating women could reach 10% of these recommendations through the daily consumption of SFSJ4.

### 3.2. Mineral Content in Commercial and Fresh Orange Juices

The content of six minerals (Ca, K, Mg, Fe, Mn, and Zn) was determined in the juice samples selected. Fresh juice samples showed significant different values of minerals in comparison to FC (except for Mg) and NFC samples (except for Ca and Mg). On the other hand, no significant differences were found in the mineral content of FC and NFC samples, except for Ca levels (Table 5).

Regarding the commercial orange juice samples, the mean contents of Ca, K, Mg, Mn, and Zn were significantly different among purchasing countries, whereas the concentration of Fe was not significantly different among countries, nor among juice samples sold in the same country in the case of UK and Germany samples (*p* > 0.05).

Orange juices sold in Germany, Spain, and France reported the highest contents of Ca (8.43–14.22 mg/100 mL), Fe (0.082–0.375 mg/100 mL), and Zn (0.042–0.071 mg/100 mL), respectively. The concentrations of K, Mg, and Mn were generally higher in juices sold in the UK (187.66–220.65 mg K/100 mL, 10.22–12.33 mg Mg/100 mL, and 0.033–0.051 mg Mn/100 mL, respectively).

Focusing on individual commercial juices, two samples sold in France (FJ1 NFC and FJ5 FC) showed the highest values for K and Zn (223.40 mg K/100 mL and 0.071 mg Zn/100 mL, respectively), one sample sold in the UK (UKJ1 NFC) reported the highest value for Mg (12.33 mg/100 mL), two FC samples sold in Germany (GJ4 FC and GJ5F C) had the highest concentrations of Ca and Mn (14.22 mg Ca/100 mL and 0.055 mg Mn/100 mL, respectively), and one sample sold in Spain (SJ3 NFC) had the highest Fe content (0.375 mg Fe/100 mL).

According to Berk (2016) [21], citrus juices are a fairly good source of minerals, particularly K, with typical values of around 200 mg/mL in orange juice. Concerning the fresh orange juices (FSJ) assessed in this study, SFSJ4 had the highest values for Ca (7.64 mg/100 mL), Mn (0.024 mg/100 mL), and Zn (0.043 mg/100 mL), whereas SFSJ1 had the highest content of K (197.09 mg/100 mL) and Mg (11.12 mg/100 mL), and SFSJ3 juice had the maximum Fe concentration (0.078 mg/100 mL). However, fresh orange juices had lower mineral concentrations in comparison with commercial juices (Table 5 and Table 6). Differences could be related to the different extraction procedures likely used among samples [22].

Among the FSJ samples, storage time had no significant effect (*p* > 0.05) on mineral content (t = 0–48 h) (Table 6), except for SFSJ1, which showed significant reductions in the concentration of K and Zn, and SFSJ2 and SFSJ3, which showed a reduction in the concentration of Mn.

#### Orange Juice Contribution to Potassium Requirements

Commercial and fresh juices analyzed contained higher amounts of K than orange juices considered by other authors. Dehelean and Magdas (2013) and Schmutzer et al. (2016) analyzed different commercial orange juices and reported K contents of 7.29–64.23 mg/100 mL and 7.57–147.17 mg/100 mL, respectively [23,24]. Fresh orange juices analyzed by Topuz et al. (2005) showed lower 101.1–136.4 mg K/100 mL, a lower concentration of this mineral in comparison with the samples selected for this study [25].

Following the EFSA report on the daily nutritional requirements recommended for different target groups of the population [20], the consumption of the orange juices analyzed in the present study could contribute to the achievement of the daily requirements of potassium established for children, teenagers, and adults, as well as vulnerable groups such as pregnant and lactating women. As it can be deduced from the information presented in Table 7, the consumption of only one glass of commercial orange juice (200 mL) could contribute to at least 40% of K daily requirements in toddlers between 1 and 3 years old, as well as 30% and 20% in children between 4 and 6 years old and 7 and 10 years, respectively. Teenagers (11–17 years) would also benefit from the intake of one glass of all analyzed orange juices, as they could cover 10–15% of the daily requirements of this mineral. Considering those samples with the highest content of K, that is, juices sold in the UK and Germany, it can be concluded that almost 11% of K requirements could be fulfilled in the general population (>18 years), including pregnant and lactating women.

Regarding the fresh orange juices, contributions to K requirements were interestingly similar to the commercial samples. For instance, one glass of these fresh juices could meet at least 40% in toddlers 1–3 years old, whereas in children 4–10 years, their contribution ranged between 17.6 and 35.8%. In the case of teenagers (11–17 years old), they could benefit from the intake of these fresh orange juices as their K requirements could be reached from 9 to 14.6%.

### 3.3. Potential Nutrition Claims

In terms of labeling, and according to the European Regulation in force (Regulation (EC) Nº 1924/2006 and Regulation (EU) Nº 1169/2011)—also adopted in the UK—analyzed orange juices, especially fresh samples, showed interesting contents of folate. According to our analysis, commercial juices NFC met the conditions of use for the nutrition claim: “Source of folates” with independence of being sold in France (FJ1 NFC, FJ2 NFC, FJ3 NFC, FJ4 NFC) and in the UK (UKJ1 NFC, UKJ2 NFC, UKJ3 NFC, UKJ4 NFC), as well as FC sample (FJ5 FC). In addition, fresh juice (FSJ) samples SFSJ1 and SFSJ4 met the conditions of use for a “Source of folates” claim. Nutrition claims can be used in labeling and other commercial communications as well as enable access to related health claims for folate provided by the European Commission.

Considering the relevant amounts of potassium in orange juices analyzed and under the European regulation in force (Regulation (EC) Nº 1924/2006, Regulation (EU) Nº 1169/2011), all commercial and fresh orange juices could use the nutrition claim “Source of potassium” in commercial communications and access associated health claims.

## 4. Conclusions

Folate concentration was highly variable between commercial samples, with higher contents corresponding to FSJ and NFC samples, followed by FC juices. Regarding mineral content, FSJs showed significant differences with FC (except for Mg) and NFC juice samples (except for Ca and Mg), while FC and NFC had similar mineral profiles, except for Ca levels. Storage time had no significant impact on FSJ folates and minerals. Among commercial orange juices, the NFC category generally showed the highest content of folates, K, Mg, and Fe, whereas juices made from concentrate showed the highest contents of Ca, Mn, and Zn.

The consumption of the analyzed orange juices could interestingly contribute to the achievement of the daily requirements of essential compounds like folates and potassium, particularly in children and teenagers, the most benefited population groups. Finally, some commercial juices could be considered a “Source of folate”, whereas all samples (commercial and fresh juices) could use the nutrition claim “Source of potassium” in their labeling, presentation, and/or advertising, according to the European regulation in force.

## Figures and Tables

**Table 1:** Description of fresh and commercial orange juices analyzed in this study.

Freshly Squeezed Juices (FSJ)		
***Code sample***	***Origin***	***Stored***
SFSJ1	Spain	Chilled/Refrigeration
SFSJ2	Spain	Chilled/Refrigeration
SFSJ3	Spain	Chilled/Refrigeration
SFSJ4	Spain	Chilled/Refrigeration
**Not From Concentrate (NFC)**		
***Code sample***	***Retail location***	***Stored***
FJ1 NFC	France	Chilled/Refrigeration
FJ2 NFC	France	Chilled/Refrigeration
FJ3 NFC	France	Room temperature
FJ4 NFC	France	Chilled/Refrigeration
UKJ1 NFC	UK	Chilled/Refrigeration
UKJ2 NFC	UK	Chilled/Refrigeration
UKJ3 NFC	UK	Chilled/Refrigeration
UKJ4 NFC	UK	Chilled/Refrigeration
SJ3 NFC	Spain	Chilled/Refrigeration
SJ4 NFC	Spain	Chilled/Refrigeration
**From Concentrate (FC)**		
FJ5 FC	France	Room temperature
UKJ5 FC	UK	Room temperature
UKJ6 FC	UK	Room temperature
GJ1 FC	Germany	Room temperature
GJ2 FC	Germany	Room temperature
GJ3 FC	Germany	Room temperature
GJ4 FC	Germany	Room temperature
GJ5 FC	Germany	Room temperature
SJ1 FC	Spain	Room temperature
SJ2 FC	Spain	Room temperature
SJ5 FC	Spain	Room temperature

**Table 2:** Folic acid (5-MTHF) content in fresh and commercial orange juice samples.

	5-MTHF (µg/100 mL)
Differences between processing techniques, ANOVA Test	Significance
FSJ–NFC	NS
FSJ–FC	*
FC–NFC	*
Differences between processing techniques, ANOVA Test	Significance
France	***
UK	***
Germany	***
Spain	***
Countries	***
Tukey’s Multiple Range Test +	
Spanish Fresh Squeezed Juices (t = 0)	5-MTHF (µg/100 mL)
SFSJ1	23.860 (0.530) ^c^
SFSJ2	6.025 (0.113) ^a^
SFSJ3	7.787 (0.055) ^b^
SFSJ4	25.510 (1.594) ^d^
Commercial orange juices	Mean
France	
FJ1 NFC	17.253 (0.075) ^c^
FJ2 NFC	23.193 (0.681) ^a^
FJ3 NFC	20.030 (0.881) ^b^
FJ4 NFC	19.966 (0.766) ^b^
FJ5 FC	17.323 (0.260) ^c^
UK	
UKJ1 NFC	16.500 (0.766) ^c^
UKJ2 NFC	18.063 (0.826) ^b^
UKJ3 NFC	21.130 (0.121) ^a^
UKJ4 NFC	18.917 (0.051) ^b^
UKJ5 FC	13.880 (0.154) ^d^
UKJ6 FC	13.447 (0.291) ^d^
Germany	
GJ1 FC	3.850 (0.227) ^b^
GJ2 FC	4.390 (0.173) ^b^
GJ3 FC	3.887 (0.057) ^b^
GJ4 FC	4.817 (0.109) ^b^
GJ5 FC	6.790 (0.060) ^a^
Spain	
SJ1 FC	8.252 (0.287) ^b^
SJ2 FC	8.250 (0.040) ^b^
SJ3 NFC	8.150 (0.175) ^b^
SJ4 NFC	8.360 (0.177) ^b^
SJ5 FC	11.380 (0.701) ^a^

Note: NS: not significant at *p* > 0.05; * and *** significant at *p* < 0.05 and 0.001, respectively. + Values: the mean of three replications (standard deviations), followed by the same letter within the same column and country, were not significantly different (*p* > 0.05). Results were expressed as µg 5-MTHF/100 mL.

**Table 3:** Folic acid (5-MTHF) content in fresh orange juice samples, from Spain, at four time points (t = 0, 12, 24, and 48 h).

	Samples			
Time	SFSJ1	SFSJ2	SFSJ3	SFSJ4
t0	23.860 (0.530) ^a^	6.025 (0.113) ^a^	7.787 (0.055) ^a^	25.510 (1.592) ^ab^
t12	21.243 (0.925) ^a^	5.733 (0.593) ^a^	7.343 (0.461) ^a^	23.307 (0.540) ^b^
t24	24.580 (0.454) ^a^	5.547 (0.481) ^a^	7.123 (0.071) ^a^	25.883 (0.200) ^a^
t48	18.897 (0.681) ^a^	5.660 (0.440) ^a^	6.920 (0.503) ^a^	26.573 (0.690) ^a^
**ANOVA Test**	NS	NS	NS	**

Note: The value mean of three replications (standard deviations) followed by the same letter within the same column and country were not significantly different (*p* > 0.05). NS: not significant at *p* > 0.05; ** significant at *p* < 0.001. Results were expressed as µg 5-MTHF/100 mL.

**Table 4:** Contribution of commercial and fresh orange juices (mean value of t = 0) to folate daily requirements established for different target groups (children aged 1–3 years, 4–6 years, and 7–10 years); teenagers (aged 11–14 years and 15–17 years); >18 years; pregnant women; and lactating women [20].

Age	Sex	5-MTHF	% Contribution				% Contribution			
		μg/day	France	UK	Spain	Germany	SFSJ1	SFSJ2	SFSJ3	SFSJ4
1–3 years	-	120	32.6	28.3	14.8	7.9	39.8	10.0	13.0	42.5
4–5 years	-	150	26.1	22.7	11.8	6.3	31.8	8.0	10.4	34.0
6–9 years	-	200	19.6	17.0	8.9	4.8	23.9	6.0	7.8	25.5
10–13 years	Men	270	14.5	12.6	6.6	3.5	17.7	4.5	5.8	18.9
	Women	270	14.5	12.6	6.6	3.5	17.7	4.5	5.8	18.9
14–19 years	Men	330	11.9	10.3	5.4	2.9	14.5	3.7	4.7	15.5
	Women	330	11.9	10.3	5.4	2.9	14.5	3.7	4.7	15.5
20–70 years	Men	330	11.9	10.3	5.4	2.9	14.5	3.7	4.7	15.5
	Women	330	11.9	10.3	5.4	2.9	14.5	3.7	4.7	15.5
-	Pregnant women	500	7.8	6.8	3.6	1.9	9.5	2.4	3.1	10.2
-	Lactating women	500	7.8	6.8	3.6	1.9	9.5	2.4	3.1	10.2

**Table 5:** Mean mineral content of the most relevant minerals (Ca, K, Mg, Fe, Mn, and Zn) present in commercial and fresh orange juice samples. Results expressed as mg/100 mL.

Samples	Ca	K	Mg	Fe	Mn	Zn
ANOVA Tests among different types of juices						
FC-FSJ	*	*	NS	*	*	*
FC-NFC	*	NS	NS	NS	NS	NS
FSJ-NFC	NS	*	NS	*	*	*
ANOVA Tests within and among countries						
France						
FJ1 NFC	10.648 (0.922) ^a^	223.404 (6.618) ^a^	12.081 (0.440) ^a^	0.171 (0.029) ^a^	0.043 (0.007) ^a^	0.056 (0.003) ^abc^
FJ2 NFC	7.479 (0.170) ^bc^	177.086 (0.265) ^b^	10.372 (0.010) ^cd^	0.087 (0.006) ^b^	0.026 (0.000) ^c^	0.042 (0.000) ^a^
FJ3 NFC	6.298 (0.037) ^c^	185.696 (2.787) ^b^	9.966 (0.149) ^d^	0.106 (0.001) ^b^	0.035 (0.002) ^ab^	0.065 (0.015) ^bc^
FJ4 NFC	8.856 (0.749) ^b^	185.141 (2.324) ^b^	11.263 (0.208) ^b^	0.113 (0.004) ^b^	0.043 (0.002) ^a^	0.046 (0.001) ^ab^
FJ5 FC	8.246 (0.178) ^b^	181.302 (7.265) ^b^	10.831 (0.210) ^bc^	0.118 (0.008) ^b^	0.032 (0.001) ^bc^	0.071 (0.024) ^c^
ANOVA test	***	***	***	***	***	NS
UK						
UKJ1 NFC	8.908 (0.055) ^b^	220.648 (3.052) ^a^	12.329 (0.191) ^a^	0.137 (0.013) ^ab^	0.051 (0.000) ^a^	0.052 (0.001) ^a^
UKJ2 NFC	8.763 (0.083) ^b^	209.656 (1.792) ^ab^	11.915 (0.118) ^ab^	0.132 (0.027) ^ab^	0.046 (0.002) ^bc^	0.045 (0.001) ^ab^
UKJ3 NFC	6.382 (0.005) ^d^	187.662 (0.235) ^c^	10.221 (0.036) ^d^	0.095 (0.009) ^a^	0.033 (0.003) ^d^	0.041 (0.000) ^b^
UKJ4 NFC	7.148 (0.044) ^c^	198.417 (5.359) ^bc^	10.976 (0.293) ^c^	0.144 (0.050) ^b^	0.043 (0.001) ^bc^	0.044 (0.004) ^ab^
UKJ5 FC	11.949 (0.228) ^a^	198.957 (11.052) ^bc^	11.505 (0.077) ^bc^	0.126 (0.030) ^ab^	0.047 (0.002) ^ab^	0.041 (0.008) ^b^
UKJ6 FC	11.918 (0.447) ^a^	201.796 (4.862) ^bc^	11.201 (0.285) ^c^	0.097 (0.002) ^ab^	0.042 (0.001) ^c^	0.047 (0.001) ^ab^
ANOVA test	***	***	***	NS	***	**
Germany						
GJ1 FC	10.622 (0.199) ^bc^	202.755 (2.845) ^a^	11.859 (0.191) ^a^	0.126 (0.020) ^a^	0.041 (0.000) ^bc^	0.051 (0.001) ^a^
GJ2 FC	8.432 (0.290) ^c^	189.914 (6.703) ^bc^	10.462 (0.784) ^b^	0.149 (0.011) ^a^	0.032 (0.009) ^c^	0.042 (0.002) ^b^
GJ3 FC	12.751 (0.032) ^ab^	197.339 (1.044) ^ab^	11.215 (0.051) ^ab^	0.116 (0.006) ^a^	0.036 (0.001) ^c^	0.046 (0.002) ^ab^
GJ4 FC	14.222 (1.932) ^a^	193.217 (4.635) ^ab^	12.019 (0.650) ^a^	0.119 (0.020) ^a^	0.048 (0.001) ^ab^	0.049 (0.004) ^ab^
GJ5 FC	10.307 (0.005) ^c^	181.455 (1.333) ^c^	11.391 (0.076) ^ab^	0.135 (0.046) ^a^	0.055 (0.001) ^a^	0.044 (0.004) ^ab^
ANOVA test	***	***	**	NS	***	**
Spain						
SJ1 FC	5.473 (0.241) ^c^	167.720 (5.813) ^b^	8.943 (0.318) ^b^	0.085 (0.006) ^c^	0.030 (0.002) ^b^	0.042 (0.001) ^a^
SJ2 FC	6.723 (0.143) ^b^	189.997 (5.080) ^a^	9.629 (0.280) ^ab^	0.104 (0.007) ^c^	0.023 (0.002) ^c^	0.037 (0.002) ^b^
SJ3 NFC	6.852 (0.130) ^b^	168.774 (3.647) ^b^	9.050 (0.207) ^ab^	0.375 (0.054) ^a^	0.025 (0.001) ^c^	0.039 (0.000) ^ab^
SJ4 NFC	9.332 (0.254) ^a^	178.733 (1.426) ^ab^	10.261 (0.057) ^a^	0.082 (0.001) ^c^	0.018 (0.001) ^d^	0.036 (0.001) ^b^
SJ5 FC	9.385 (0.635) ^a^	173.173 (15.282) ^ab^	9.986 (0.925) ^ab^	0.227 (0.038) ^b^	0.042 (0.003) ^a^	0.040 (0.003) ^ab^
ANOVA test	***	**	**	***	***	**
ANOVA test among countries of purchasing	***	***	***	NS	***	***

Note: NS: not significant at *p* > 0.05; *, **, and *** significant at *p* < 0.05, 0.01, and 0.001, respectively. Values: the mean of three replications (standard deviations) followed by the same letter within the same column and country were not significantly different (*p* > 0.05).

**Table 6:** The mean mineral content of fresh orange juice samples as obtained at t0 after storage (t = 0 to 48 h). Results expressed as mg/100 mL.

Samples at t = 0	Ca	K	Mg	Fe	Mn	Zn
SFSJ1	7.330 (0.053) ^b^	197.088 (1.888) ^c^	11.017 (0.139) ^b^	0.069 (0.011) ^a^	0.012 (0.002) ^a^	0.036 (0.000) ^b^
SFSJ2	7.182 (0.584) ^b^	182.222 (12.752) ^b^	9.881 (0.391) ^a^	0.076 (0.012) ^a^	0.021 (0.000) ^b^	0.032 (0.000) ^a^
SFSJ3	5.899 (0.602) ^a^	158.362 (2.573) ^a^	10.515 (0.171) ^ab^	0.078 (0.001) ^a^	0.012 (0.001) ^a^	0.036 (0.002) ^b^
SFSJ4	7.644 (0.130) ^b^	163.459 (4.243) ^a^	9.963 (0.294) ^a^	0.077 (0.002) ^a^	0.024 (0.003) ^b^	0.043 (0.001) ^c^
**ANOVA Test—Influence of Storage time (from 0 to 48 h)**						
**Samples**	**Ca**	**K**	**Mg**	**Fe**	**Mn**	**Zn**
SFSJ1	NS	**	NS	NS	NS	***
SFSJ2	NS	NS	NS	NS	***	NS
SFSJ3	NS	NS	NS	NS	**	NS
SFSJ4	NS	NS	NS	NS	NS	NS

Note: NS: not significant at *p* > 0.05; ** and *** significant at *p* < 0.01 and 0.001, respectively. Values: the mean of three replications (standard deviations) followed by the same letter were not significantly different (*p* > 0.05).

**Table 7:** Contribution of commercial and fresh orange juices (mean value of t = 0) to potassium daily requirements established for different target groups (children aged 1–3 years, 4–6 years, and 7–10 years); teenagers (aged 11–14 years and 15–17 years); >18 years; pregnant women; and lactating women [20].

Age	Sex	Potassium Requirements	% Contribution of Commercial Orange Juices from Different Retail Locations				% Contribution of Fresh Orange Juices			
		mg/day	France	UK	Spain	Germany	SFSJ1	SFSJ2	SFSJ3	SFSJ4
1–3 years	-	800	47.6	50.7	43.9	48.2	49.3	45.6	39.6	40.9
4–6 years	-	1100	34.6	36.9	31.9	35.1	35.8	33.1	28.8	29.7
7–10 years	-	1800	21.2	22.5	19.5	21.4	21.9	20.2	17.6	18.2
11–14 years	Men	2700	14.1	15.0	13.0	14.3	14.6	13.5	11.7	12.1
	Women	2700	14.1	15.0	13.0	14.3	14.6	13.5	11.7	12.1
15–17 years	Men	3500	10.9	11.6	10.0	11.0	11.3	10.4	9.0	9.3
	Women	3500	10.9	11.6	10.0	11.0	11.3	10.4	9.0	9.3
>18 years	Men	3500	10.9	11.6	10.0	11.0	11.3	10.4	9.0	9.3
	Women	3500	10.9	11.6	10.0	11.0	11.3	10.4	9.0	9.3
-	Pregnant women	3500	10.9	11.6	10.0	11.0	11.3	10.4	9.0	9.3
-	Lactating women	4000	9.5	10.1	8.8	9.6	9.9	9.1	7.9	8.2

## Data Availability

The original contributions presented in the study are included in the article, further inquiries can be directed to the corresponding author.
